# Cyclic Fevers in Pregnancy: A Case of Parvovirus‐Related Hemophagocytic Lymphohistiocytosis

**DOI:** 10.1155/crog/7218689

**Published:** 2026-06-10

**Authors:** Dominick J. Rich, Meridith P. Pollie, Julie P. Barbera, Jordan S. Stone, Adi Hirshberg

**Affiliations:** ^1^ Department of Obstetrics and Gynecology, Perelman School of Medicine at the University of Pennsylvania, Philadelphia, Pennsylvania, USA, upenn.edu; ^2^ Department of Obstetrics and Gynecology, University of Pennsylvania Health System, Philadelphia, Pennsylvania, USA, pennmedicine.org

**Keywords:** hemophagocytic lymphohistiocytosis, parvovirus, sickle cell disease

## Abstract

Parvovirus B19 typically causes mild symptoms in healthy adults, including fever, rash, and arthralgias. Certain immunocompromised adult populations, including those with immunodeficiencies or hematologic disease, are at risk of clinically significant complications, including transient aplastic crisis. Further, in pregnant patients, there is a risk of transplacental viral transmission, which may lead to severe fetal anemia, nonimmune hydrops fetalis, and intrauterine fetal demise. The clinical course of parvovirus B19 in pregnant patients with underlying hematologic disease has not been well reported. In this case report, we present a 27‐year‐old pregnant patient with sickle cell beta thalassemia who was infected with parvovirus B19 at 20 weeks of gestation. She experienced a prolonged hospitalization characterized by persistent maternal vaso‐occlusive crisis with concomitant transient aplastic crisis. There was suspected vertical transmission of the virus, leading to concern for severe fetal anemia and termination of the pregnancy. The patient continued to have cyclic fevers, bicytopenia, and a constellation of laboratory findings leading to a diagnosis of postviral hemophagocytic lymphohistiocytosis (HLH). She was successfully treated with pulse dose corticosteroids and an Interleukin‐1 receptor antagonist with subsequent improvement of her symptoms and laboratory markers. This report highlights the distinct diagnostic and management challenges of parvovirus B19 in pregnant patients with comorbid hematologic disease, including a rare but serious sequela of the infection, and the risk for severe maternal and fetal complications.

## 1. Introduction

Parvovirus B19 can cause acute infection in adult populations. While the pathognomonic presentation in pediatric patients includes flu‐like symptoms often followed by a “slapped cheek” rash, parvovirus B19 is often asymptomatic in healthy adults [1, 2]. However, certain conditions place adult populations at risk of clinically significant disease, including immunocompromise, pregnancy, or comorbid hematologic disease [2–4]. In these patients, the clinical manifestations of parvovirus infection range from a rash with mild respiratory symptoms to fever and polyarthralgia with transient aplastic crisis (TAC) [2–5]

Approximately 35% of American women are nonimmune to parvovirus B19 at the beginning of pregnancy, with an incidence of seroconversion in pregnancy estimated to be 1% [6]. The risk of transplacental viral transmission ranges from 17% to 33% and can lead to adverse fetal outcomes, including anemia, hydrops fetalis, and intrauterine fetal demise [7–10]. Low‐risk pregnant people with acute parvovirus B19 infection will be asymptomatic in 30%–50% of cases. Symptomatic patients commonly present with arthralgia and fevers [9–11]. However, the disease manifestations and clinical course of infection in pregnant patients with additional high‐risk comorbidities have not been well reported. A significant gap exists in the current literature surrounding the diagnostic uncertainty and management options of parvovirus‐related hemophagocytic lymphohistiocytosis (HLH) in pregnant patients, especially in the setting of concomitant vaso‐occlusive crisis (VOC). Here, we present the case of a pregnant patient with sickle cell beta thalassemia who developed acute parvovirus B19 infection leading to severe maternal and fetal complications.

## 2. Case Presentation

### 2.1. Presentation and Admission

Our patient is a 27‐year‐old G3P2002 with sickle cell beta thalassemia and multiple prior VOCs. She initially presented at 20 weeks 4 days of gestation with unilateral hip and rib pain. She denied any respiratory, gastrointestinal, or urinary complaints. She was found to be tachycardic to 112 beats per minute and febrile to 101.3 F. Initial laboratory assessment on Hospital Day (HD) 0 showed elevated lactate dehydrogenase to 649 U/L, but her other labs were unremarkable, including a white blood cell count of 4.7∗10^3^/*μ*L, platelets of 191∗10^3^/*μ*L, hemoglobin near her baseline at 9.0 g/dL, reticulocyte count of 1.3%, and haptoglobin of 133 mg/dL. Chest X‐ray, urinalysis, respiratory pathogen panel, and blood cultures were negative. She was admitted for ongoing workup and management of her fevers, pain, and laboratory abnormalities.

### 2.2. Infectious Workup

**Figure 1:**
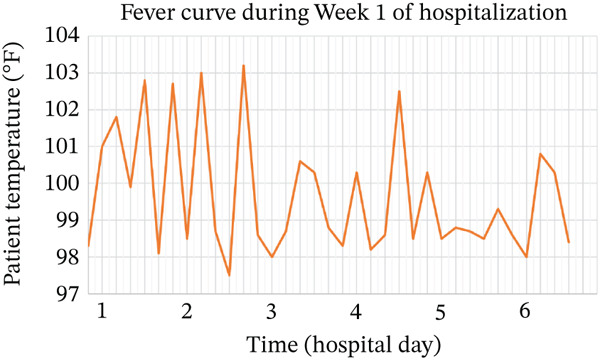
Patient temperature, reported at 4‐h intervals, during the first 7 days of her hospitalization. Twice daily fevers can be appreciated.

**Figure 2:**
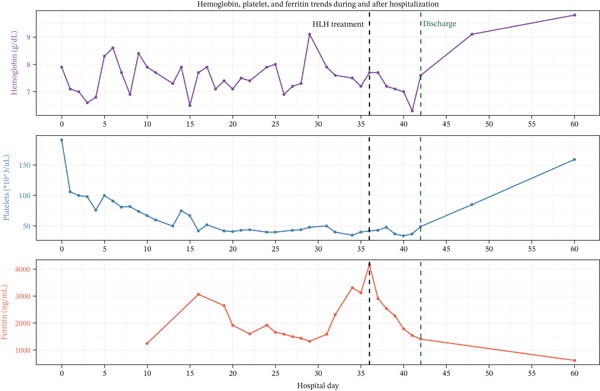
Hemoglobin concentration, platelet count, and ferritin concentration laboratory values demonstrating trends over the 42‐day admission period and initial 18‐day postdischarge period. HLH treatment initiation (black, dotted line) and discharge date (green, dotted line) are overlaid.

Shortly after admission, on HD1, the patient reported chills, rigors, and severely worsening 10/10 pain in the same locations of the right hip and ribs. She was found to be febrile to 103.8 F, and repeat laboratory tests showed decreased hemoglobin to 7.9 g/dL, haptoglobin to 93 mg/dL, and platelets to 145∗10^3^/*μ*L, with sickling of red blood cells (RBC) on peripheral smear (Table S1). She was started on intravenous fluids and opioids for VOC, and the infectious disease team was consulted due to concern for underlying infection on the basis of her atypical crisis presentation with fevers and bicytopenia. On HD3, a diagnostic workup was initiated by infectious disease, including negative repeat blood and urine cultures (no growth); polymerase chain reaction (PCR) testing for EBV, HIV, and CMV (all nondetectable); repeat CXR (clear); and CTPE (no evidence of pulmonary embolus and incidental possible bronchitis). Notably, parvovirus B19 serologies and serum DNA PCR resulted in HD6 with positive B19 IgG of 8.19 IV, negative IgM of 0.23 IV, and B19 DNA of 354 IU/mL by PCR. At that time, hemoglobin electrophoresis demonstrated Hemoglobin A 41.8%, Hemoglobin A2 4.5%, Hemoglobin F 3.3%, and Hemoglobin S 50.4%, consistent with prior testing. Over the subsequent 2 weeks, she continued to have twice‐daily fevers at nearly 12‐h intervals despite scheduled dosing of antipyretics (Figure 1). Her daily labs demonstrated worsening leukopenia (nadir 2.6∗10^3^/*μ*L), thrombocytopenia (nadir 43∗10^3^/*μ*L), and anemia with a reticulocyte count persistently < 2.5% (Figure 2). She consistently reported severe 10/10 pain during febrile episodes, and as a result, multiple attempts to transition from an intravenous hydromorphone patient–controlled anesthesia to oral medications were unsuccessful.

### 2.3. Fetal Management

Given the parvovirus infection, monitoring was initiated for signs of fetal anemia with fetal middle cerebral artery (MCA) dopplers. On HD9, 3 days after serologic confirmation of parvovirus B19 infection, initial MCA peak systolic velocity (MCA‐PSV) measurements demonstrated mild fetal anemia. One week later, on HD16, repeat MCA dopplers revealed moderate to severe fetal anemia with MCA‐PSV of 44.29 cm/s (1.51 MoM). There was no evidence of hydrops fetalis on ultrasound. Although the fetus met standard criteria for percutaneous umbilical blood sampling (PUBS) and potential intrauterine transfusion (IUT) based on an MCA‐PSV of 1.51 MoM, the decision to offer fetal intervention required careful consideration of significant maternal risk factors. The patient was counseled on management options, including pregnancy continuation with PUBS and potential IUT versus termination, given her complicated clinical course and early gestational age. At the time of counseling, the patient was experiencing ongoing VOCs and persistent daily fevers, with laboratory evidence of TAC, including anemia, thrombocytopenia, and leukopenia. These conditions substantially increased the risk of maternal hemodynamic instability, procedural bleeding, and infection. Furthermore, technical challenges posed by pain, sedation requirements, and frequent febrile spikes raised concerns regarding the safety and feasibility of PUBS/IUT. In addition, the ongoing maternal inflammatory state of unclear etiology created uncertainty about the likelihood of requiring multiple fetal transfusions and the potential impact on fetal neurologic outcomes. After reviewing these risks, the patient elected pregnancy termination rather than pursuing fetal intervention and underwent dilation and evacuation on HD21 at 23 weeks 4 days. Fetal pathology after termination was consistent with products of conception and was otherwise nondiagnostic.

### 2.4. HLH Diagnosis and Management

In the postoperative period, the patient experienced minimal symptom relief. Additional autoinflammatory workup on HD16 yielded initial concern for HLH given persistent relapsing fevers with pancytopenia and newly elevated triglycerides to 277 mg/dL and ferritin to 3065 mcg/L. Prior to the completion of the HLH workup, a 2‐day trial of IVIG (1 g/kg) was initiated by the hematology team on HD21 for the leading diagnosis of persistent parvovirus infection, with no improvement in symptomatology. Soluble Interleukin‐2 receptor and Chemokine Ligand 9 markers (collected HD19, resulted HD24) were found to be markedly elevated to 5544 and 19961 pg/mL, respectively, and a bone marrow biopsy (performed HD22, finalized HD27) demonstrated rare hemophagocytic histiocytes. On further review, the patient denied any family history of primary HLH or consanguinity. At that time, her HScore was calculated as 203, indicating an 88%–93% probability of hemophagocytic syndrome [12]. However, the setting of her known sickle cell disease, which can independently result in an elevated HScore given overlapping features including hepatosplenomegaly, cytopenias, and transaminitis, resulted in interpretation challenges. The patient simultaneously reported new floaters in bilateral eyes, and ophthalmologic testing revealed bilateral retinal thickening, perivascular sheathing, and focal areas of vasculitis with intraretinal flame hemorrhage, concerning for retinal periphlebitis. She was treated for retinal vasculitis with high‐dose oral corticosteroids on HD30 (60 mg prednisone), which the rheumatology team supported could theoretically improve her persistent inflammatory state. In subsequent multidisciplinary discussions with the infectious disease, hematology, and rheumatology teams, her constellation of symptoms and laboratory abnormalities was determined to be consistent with parvovirus‐induced HLH but without a clear diagnostic consensus across involved specialists, resulting in the pursuit of nonchemoimmunotherapeutic treatment modalities for likely HLH. On HD36, she was started on 5 days of pulse‐dose IV methylprednisone 1 g, followed by prednisone 60 mg daily and anakinra 100 mg daily. Her fever, labs, and symptoms gradually improved, and she was discharged on HD42 on continued immunosuppression (Figure 2). Given her continued clinical improvement in the postdischarge setting, she has been maintained on continued immunosuppression with anakinra 100 mg and prednisone 20 mg daily for diagnosed HLH. She has had no relapse events and maintains a good prognosis.

## 3. Discussion

The natural history of parvovirus B19 infection has been well documented in the general population and in pregnancy; however, there is a gap in the literature addressing specific diagnostic and management considerations for pregnant patients with underlying hematologic disease infected with parvovirus B19. This report provides valuable information on the course of infection in this patient population.

### 3.1. Diagnostic Challenges

Arriving at a clear diagnosis can be challenging, given the overlap in signs and symptoms among VOC, parvovirus, and other inflammatory conditions in pregnant patients with hemoglobinopathies. Our patient presented with fever and joint pain, two of the most common symptoms of inflammation in pregnant and nonpregnant adults alike [3]. This nonspecific presentation requires that clinicians maintain a broad differential, including infectious and noninfectious inflammatory conditions. In a patient with hematologic disease, this is further complicated by the inflammatory overlap with VOC. Initially, our patient presented with hemoglobin below baseline and reported large joint/bone pain consistent with her prior crises, but the concomitant fever > 38°C and bicytopenia without a compensatory rise in reticulocyte count warranted further workup, eventually leading to the parvovirus B19 diagnosis. When high‐risk patients develop symptoms or laboratory findings that stray from their typical VOC presentations, clinicians should keep viral‐induced TAC on the differential.

### 3.2. Maternal–Fetal Transmission

Transplacental spread of parvovirus B19 poses significant fetal risks. The incidence of fetal anemia, nonimmune fetal hydrops, or intrauterine fetal demise is much higher when infection occurs in early pregnancy, especially between 9 and 20 weeks of gestational age, with rates of 5%–10% before 12 weeks of gestational age, 5% before 20 weeks of gestational age, and less than 1% after 20 weeks [9]. When considering screening and fetal interventions for pregnant patients infected with parvovirus B19, clinicians often use these risk profiles to help guide counseling and management.

Our patient was first symptomatic at 20 weeks, placing her in the theoretically lower risk category for maternal–fetal transmission and fetal complications. However, MCA Doppler surveillance suggested moderate to severe fetal anemia within 2 weeks of diagnosis; therefore, IUT was considered. However, decision‐making and counseling in this patient population are complicated by multiple factors. Patients with VOC and TAC may require serial IUT, compounding the risks of further infection, need for emergency C‐section, and perinatal death. Further, serial IUT may increase the likelihood of developing RBC antibodies, which can pose challenges over time to obtaining compatible RBC units for maternal transfusion in patients with sickle cell disease [13]. Lastly, fetal neurologic outcomes following daily, recurrent fevers > 39°C are uncertain, particularly when the underlying etiology and, therefore, duration of febrile morbidity are uncertain [14].

### 3.3. Serologic Discordance

Clear clinical signs and symptoms are often sufficient for the diagnosis of acute parvovirus B19 infection. However, in immunocompromised hosts lacking classic manifestations, laboratory diagnosis of parvovirus B19 is considered essential. Antibody serology, with IgM or IgG, is a widely available test but has suboptimal sensitivities due to a combination of test‐intrinsic, host, and viral factors. Therefore, B19 DNA PCR is of greatest diagnostic value in the immunocompromised patient and can be used to differentiate low‐level persistence from clinically relevant infection, especially in the setting of unclear serologic results [2, 15]. Notably, in a study of acutely infected nonpregnant patients with confirmed viremia by PCR, only 50% of patients had the expected IgG and IgM seropositivity, with others demonstrating discordant IgM and IgG serology and almost 30% being both IgG and IgM seronegative, emphasizing the importance of B19 viremia for diagnosis [15]. Our patient initially had positive IgG and negative IgM, but with detectable parvovirus B19 viremia by quantitative PCR, suggestive of acute infection. The expected duration of infection remained unclear as this patient′s course evolved. Cases of persistent parvovirus B19 infection are characterized by IgM seropositivity or the presence of DNA in the serum at 4 weeks or more from initial infection. Our patient had persistent pain, cyclic fevers, and refractory bicytopenia but had undetectable viral DNA on repeat PCR 20 days after the initial diagnosis, raising concern for a persistent inflammatory state.

### 3.4. HLH

HLH is a hyperinflammatory entity often triggered by periods of immunologic stress. It is diagnosed either by genetic confirmation of familial HLH or by fulfilling ≥ 5 of the seven HLH‐2024 criteria: (1) fever ≥ 38.5°C; (2) splenomegaly ≥ 2 cm below the costal margin; (3) ≥ 2 cytopenias of hemoglobin < 9.0 g/dL, platelets < 100 × 10^9^/L, and neutrophils < 10^9^/L; (4) fibrinogen ≤ 1.5 g/L or triglycerides ≥ 3.0 mmol/L; (5) hyperferritinemia ≥ 500 mcg/L; (6) bone marrow or other tissue confirmation of hemophagocytosis; and (7) soluble IL‐2 receptor alpha ≥ 2400 units/mL [16]. Though some of these criteria are difficult to interpret in patients with underlying hematologic conditions, our patient met all seven of the diagnostic criteria. Viral infections are known to precede HLH, with parvovirus B19 being a rare but well‐documented trigger of HLH, particularly in high‐risk patients with hemolytic disorders or immunocompromised status [17–19]. In pregnant patients, the diagnosis of HLH is associated with high maternal and fetal morbidity and mortality [20, 21]. Many cases do not resolve after delivery, requiring aggressive anti‐inflammatory treatment [21, 22]. There is increasing evidence supporting IL‐1 receptor antagonists for the treatment of HLH, and case reports have demonstrated efficacy in pregnant patients. Fetal outcomes are uncertain, with mixed reports of positive fetal outcomes and postnatal bone marrow suppression and sensorineural hearing loss [20, 21]. Other HLH treatment options, including the HLH‐94 regimen and cytotoxic drugs such as etoposide, are known to impact fetal development [20]. Our patient elected to terminate the pregnancy prior to receiving the diagnosis of HLH, but pregnant patients should be counseled on the benefits of immediate versus delayed treatment while also considering the fetal toxicity profiles of various treatment modalities. Our patient improved with a combination of pulse‐dose steroids and IL‐1 receptor antagonist, adding to the literature on postviral HLH and the use of anti‐inflammatory treatment to resolve persistent aplastic anemia and postviral hyperinflammation.

## 4. Conclusion

In conclusion, this case report adds to the limited existing literature on acute parvovirus B19 infection in pregnant patients with hematologic disease, highlighting the potential for severe maternal and fetal sequelae. We describe the unique clinical manifestations in a patient with multiple risk factors for severe morbidity and sequelae and the challenge in arriving at a clear diagnosis in patients with underlying hematologic disease. Further, we show the possibility of postviral HLH in immunocompromised pregnant patients and describe how intensive anti‐inflammatory therapy can lead to clinical improvement. Lastly, we discuss the development of suspected severe fetal complications and the uncertainty surrounding fetal and neonatal outcomes in this particular clinical circumstance, which influenced the patient′s decision to discontinue the pregnancy.

## Funding

No funding was received for this manuscript.

## Consent

Patient consent was obtained.

## Conflicts of Interest

The authors declare no conflicts of interest.

## Data Availability Statement

The authors confirm that the data supporting the findings of this study are available within this article. Institutional approval was not required for the disclosure of content in this case report. Any additional data is available from the corresponding author, DJR, upon request.

## Supporting information


**Supporting Information** Additional supporting information can be found online in the Supporting Information section. Table S1: Select patient laboratory values demonstrating trends over the 42‐day admission period and 46‐day post‐discharge period.
